# Perceived Stigma and Associated Factors among Patient with Tuberculosis, Wolaita Sodo, Ethiopia: Cross-Sectional Study

**DOI:** 10.1155/2019/5917537

**Published:** 2019-05-02

**Authors:** Bereket Duko, Asres Bedaso, Getinet Ayano, Zegeye Yohannis

**Affiliations:** ^1^Faculty of Health Sciences, College of Medicine and Health Sciences, P.O. Box 1560, Hawassa University, Hawassa, Ethiopia; ^2^Research and Training Directorate, Amanuel Mental Specialized Hospital, Addis Ababa, Ethiopia

## Abstract

**Background:**

Tuberculosis is a historically stigmatized disease and the stigma associated with it affects the institution, community, and interpersonal factors. Therefore, understanding tuberculosis-related perceived stigma has importance in improving quality of the patients.

**Objective:**

The aim of this study was to assess prevalence and factors associated with perceived stigma among patients with tuberculosis attending Wolaita Sodo University Referral Hospital, Ethiopia.

**Methods:**

Institution based cross-sectional study was conducted among a total of 417 tuberculosis patients who had treatment follow-up at TB clinics and were recruited for the study. Systematic random sampling technique was used to recruit study participants. A 12-item perceived TB stigma scale was used to assess tuberculosis-related perceived stigma. In addition, Oslo social support scale was used to assess social support related factors.

**Results:**

Prevalence of tuberculosis-related perceived stigma by using perceived tuberculosis stigma scale was 42.4%. Patients who had pulmonary TB [AOR=2.49, (95% CI: 1.24, 4.87)], being intensive phase category [AOR=1.42, (95% CI: 1.19, 2.58)], TB/HIV coinfection [AOR= 3.54, (95% CI: 1.37, 9.12)], poor social support [AOR=2.45, (95% CI: 1.18, 5.09)], and using substance (alcohol, khat and cigarette) [AOR=1.78, (95% CI: 1.28, 3.17)] were more likely to have perceived TB stigma when compared to their counter parts.

**Conclusion:**

Health education programs should be conducted to reduce TB stigma and improve patients' compliance.

## 1. Introduction

Tuberculosis (TB) is one of chronic infectious disease which is caused by* Mycobacterium tuberculosis* and becomes the leading causes of morbidity and mortality worldwide [1, 2]. It is a key public health concern in Ethiopia: in 2009/2010, it was the second most important cause of death [3]. Ethiopia is ranked seventh among the 22 high-burden countries that account for 81% of all cases of TB and 80% of all TB deaths worldwide [4]. Ethiopia is also one of the 27 countries identified as having a high prevalence of multidrug-resistant TB (MDR-TB). The burden of MDR-TB in these countries accounts for 86% of cases worldwide [3, 5].

Stigma stands as decreasing attribute which arises from social interaction and is related to the power dominance and difference [6]. Social (enacted) stigma refers to experience discrimination by other people which results from inferiority in the society while perceived stigma refers to the shame and expectation of discrimination that prevents people from talking about their experiences and sense of unworthy and guilty [7–19]. TB is highly stigmatized disease which can be experienced and felt at a different social setting like home, workplace, and community [7].

Perceived stigma has a considerable impact on health that renders patients to refuse disease and medical services through discouraging health-seeking behavior which leads to distortion of health condition making difficult to treat that increases infectivity and communicability of the disease [7–13]. It is more common in patients with tuberculosis. Some studies conducted in Egypt Chest Hospital and in southern Thailand and Nepal showed that the prevalence of perceived stigma was 41.5 % and 63.3%, respectively [20–22].

Therefore, understanding tuberculosis-related perceived stigma has importance in improving the quality of these populations. Therefore, this aimed to assess prevalence and associated factors of perceived stigma among TB patients in Ethiopia.

## 2. Methods

### 2.1. Study Setting and Population

Institution based cross-sectional study design was conducted from March 2016 to May 2016 at Wolaita Sodo University Referal Hospital, Woliata Sodo, Ethiopia. Tuberculosis patients whose ages were ≥ 18 years were included in the study, while critically ill patients were excluded from the study. Single population proportion formula was used to get the required sample size. Because there was no prior study on the subject area, to get maximum sample size we used prevalence of perceived TB stigma as 50% (P=50%), 95% CI, margin error of 5%, and 10% none-response rate; the required sample size became 424. Seven patients were excluded from the study due to critical condition of the illness. Among tuberculosis patients who had treatment follow-up TB clinics during the study period in the mentioned hospital, we had recruited 417 tuberculosis patients. Systematic random sampling technique was employed to select the study participants.

### 2.2. Data Collection

Our data collection instruments had included a structured interviewer administered questionnaires on sociodemographic characteristic which mainly focuses on age, sex, education, occupation, marital status, religious view of the study participants, and others. We have also used Oslo item 3 social support scales which is 3-item questionnaire commonly used to assess social support related issues in clinical and community settings [23]. The variable of interest (outcome variable), TB stigma felt by TB patients, was collected by 12-item perceived TB stigma scale that consisted of four-point Likert scale questions concerning perceived isolation, guilt, shame, and disclosure of their tuberculosis status. It was validated in Thailand among TB and HIV patients; its goodness-of-fit was good (TLI = 94, LFI = 0.88, and RMSEA = 0.11), internal consistency was excellent (Cronbach's alphas 0.82-0.91), and test-retest reliability was moderate. It has good psychometric properties that measure stigma associated with tuberculosis and HIV/AIDS and allow assessment of stigma from community and patient perspectives. Participants were classified as having or not having perceived stigma using the mean of the stigma variable as cut-off point [21]. This scale was adopted and translated to Amharic language and it was highly reliable in the study with Cronbach's *α* of 0.95.

### 2.3. Data Processing and Analyses

We used SPSS version 22 to analyze the data. The association of each independent variable with perceived tuberculosis stigma was examined in bivariate analysis. Those confounding variables that could potentially be associated with both TB and perceived stigma like anxiety, depression, substance use, social support, and other variables with p value less than 0.2 during bivariate analysis were entered into the multivariate analysis to identify potential confounders. A p value of less than 0.05 was considered statistically significant, and adjusted odds ratio with 95% CI was calculated to determine association.

## 3. Results

### 3.1. Sociodemographic Characteristics of the Study Participants

The study recruited a total of 417 tuberculosis patients; the mean (±SD) age of the respondents was 32.3 years (±9.23). Concerning sociodemographic characteristics, 241 (57.8 %) were male, 189 (45.3%) were from protestant religious background, and 199 (47.7 %) were never married. In addition, 287 (68.8 %) patients were diagnosed with pulmonary TB, 270 (64.9 %) were in intensive phase of TB treatment, and 229 (54.9%) had good social support (Table 1).

### 3.2. Prevalence of Perceived Stigma and Associated Factors among the Study Participants

Prevalence of tuberculosis-related perceived stigma by using Perceived Tuberculosis stigma scale was 42.4%. Prevalence of isolation, guilt, disclosure, and their relationship to social support is 42.4 %, 37.9%, 40.1%, and 36.6 %, respectively. Using of binary logistic regression analysis revealed that having pulmonary TB (AOR=2.49, CI:(1.24, 4.87), (P<0.01)), being in intensive phase of tuberculosis treatment (AOR=1.42, CI: (1.19, 2.58), (P=0.02)), having comorbid HIV illness (AOR=3.54, CI:(1.37, 9.12), (P <0.01)), having poor social support (AOR=2.45,CI: (1.18, 5.09) (P <0.01)), and current substance use (AOR=1.78, CI:(1.28, 3.17), (P = 0.01)) (Table 2).

## 4. Discussion

Prevalence of TB related perceived stigma in the current study was 42.4% (95%, CI: 39.28-45.52). This finding is in agreement with study conducted in Egypt Chest Hospital and southern Thailand [20, 21]. Nevertheless, a study result was lower than study conducted in Nepal [22]. This difference might be related to variation in study design, data collection tool, sample size, and study participant's variation. The difference also attributed due to variation in culture which is supported by findings from a systematic review and other studies that revealed cultural variations is potential for stigma [24–26].

Perceived stigma was mostly prevalent in patients with pulmonary tuberculosis when compared to those who had extra pulmonary tuberculosis. In addition to this, there was significant association between TB related perceived stigma and intensive phase of treatment. This finding is in agreement with the other study [22]. TB related perceived stigma is often a product of exaggerated notions of contagiousness. Community awareness and patient education may help to mitigate the isolation and rejection of TB patients and encourage TB suspects to seek initial care.

Tuberculosis patients who had comorbid HIV infection were more likely to have perceived stigma. This might be evidenced by having diagnosed with HIV illness by itself associated with high levels of stigma. Hence, TB/HIV coinfected patients can be at higher risk of having perceived stigma [27, 28].

The study also showed that those patients who had poor social support were significantly associated with perceived stigma. Having poor social support and somatic illness may lead to increased psychological distress [29]. Tuberculosis patients who had family history of mental illness were more likely to have perceived stigma. This finding was consistent with other findings [20, 30]. This might be due to the fact that anxious patients are more prone to use substances like alcohol and cigarette to relief themselves from the internalized stigma.

Study conducted in Nigeria among patients with pulmonary tuberculosis showed that no formal education and patients who are in the working age group of 20 to 50 years had perceived TB stigma. Nevertheless, in this study there is no statistically significant association.

## 5. Conclusion

Prevalence of Tuberculosis-related perceived stigma by using perceived tuberculosis stigma scale was high (42.4%). Having pulmonary TB, being in intensive phase of tuberculosis treatment, having comorbid HIV illness, having poor social support, and using substances like alcohol and cigarette were associated with perceived stigma. Health professionals who are working TB clinics should give more emphasis to their patients and giving psychosocial counseling daily basis is recommended. Health education programs should be conducted to reduce TB stigma and improve patients' compliance.

## 6. Limitation of the Study

This study did not do detailed validation study for perceived HIV related-stigma scale and Oslo 3-item social support scale. We did not include BMI in our assessment.

## Figures and Tables

**Table 1:** Sociodemographic and clinical characteristics of TB patients on follow-up at Wolaita Sodo University Hospital, Wolaita Sodo, Ethiopia, 2016.

Variables		Frequency	Percent (%)

Age	18-24 years	96	21
	25-49 years	226	70
	>= 50 years	38	9
Sex	Male	241	58
	Female	176	42
Marital status	Married	182	44
	Single	199	48
	Divorced/Widowed	66	9
Education status	No formal education	78	19
	Grade 1-8	129	31
	Grade 9-12	116	28
	College and above	94	23
Duration of illness	< 6 months	25	6
	6 – 12 months	247	59
	>= 12 months	145	35
Phase of treatment	Intensive phase	270	65
	Continuation phase	147	35
Co-morbid chronic illness	HIV/AIDS	49	12
	Diabetes/Hypertension	24	6
	No co-morbid illness	344	83
Social support	Good	229	55
	Poor	188	45
Depressive symptoms	Yes	181	43
	No	236	57
Anxiety symptoms	Yes	173	41
	No	244	59
Substance (khat, cigarette & alcohol) use	Yes	30	7
	No	387	93

**Table 2:** Factors associated with TB related perceived stigma among patients with TB at Wolaita Sodo University Referral Hospital, Wolaita Sodo, Ethiopia, 2016, n=417.

Explanatory Variables		Perceived TB stigma		COR,95% (CI)	AOR,95% (CI)
		Yes	No		
Age	18-24	23	65	0.21, (0.09, 1.47)	
	25-49	130	161	0.47, (0.23, 1.03)	
	50 and above	24	14	*1*	*1*
Sex	Male	96	145	*1*	*1*
	Female	81	95	1.29, (1.09, 2.97)	
Educational status	No formal education	45	33	1.97, (1.07, 3.63)	
	Grade 1-8	48	81	0.84, (0.50, 1.48)	
	Grade 9-12	45	71	0.94, (0.54, 1.63)	
	College and above	39	55	*1*	*1*
Job	Civil servant	33	43	*1*	*1*
	Non-governmental	33	52	1.59, (0.84, 2.99)	
	Merchant	23	28	1.31, (0.71, 2.41)	
	Farmer	8	7	1.70, (0.84, 3.45)	
	House wives	27	25	2.37, (0.78, 7.15)	
	Jobless/Daily labors	29	60	4.35, (2.08, 8.11)	
Classification	Pulmonary TB	147	140	3.50, (2.19, 5.56)	*2.49, (1.24, 4.87) *∗∗
	Extra pulmonary TB	30	100	*1*	*1*
Phase of treatment	Intensive phase	75	134	1.97, (1.29, 4.56)	*1.42, (1.19, 2.58) *∗
	Continuous phase	102	106	*1*	*1*
Co-morbid chronic illness	HIV/AIDS	36	13	4.85, (2.48, 9.49)	*3.54, (1.37, 9.12) *∗∗
	Diabetes/Hypertension	16	8	3.50, (1.45, 8.42)	
	No chronic illness	177	219	*1*	*1*
Social support	Good	42	187	*1*	*1*
	Poor	135	53	11.34, (7.15, 17.99)	*2.45, (1.18, 5.09) *∗∗
Substance (Cigarette, khat and alcohol) use	Yes	51	28	2.87, (2.12, 4.18)	*1.78, (1.28, 3.17) *∗
	No	131	207	*1*	*1*

## Data Availability

The data used to support the findings of this study are included within the article.
